# Applying Bayesian Belief Networks to Assess Alpine Grassland Degradation Risks: A Case Study in Northwest Sichuan, China

**DOI:** 10.3389/fpls.2021.773759

**Published:** 2021-11-04

**Authors:** Shuang Zhou, Li Peng

**Affiliations:** ^1^Research Center for Mountain Development, Institute of Mountain Hazards and Environment, Chinese Academy of Sciences, Chengdu, China; ^2^College of Resources and Environment, University of Chinese Academy of Sciences, Beijing, China; ^3^College of Geography and Resources, Sichuan Normal University, Chengdu, China

**Keywords:** Bayesian belief networks, alpine grassland degradation, frequency ratio model, NDVI, risk assessment

## Abstract

Grasslands are crucial components of ecosystems. In recent years, owing to certain natural and socio-economic factors, alpine grassland ecosystems have experienced significant degradation. This study integrated the frequency ratio model (FR) and Bayesian belief networks (BBN) for grassland degradation risk assessment to mitigate several issues found in previous studies. Firstly, the identification of non-encroached degraded grasslands and shrub-encroached grasslands could help stakeholders more accurately understand the status of different types of alpine grassland degradation. In addition, the index discretization method based on the FR model can more accurately ascertain the relationship between grassland degradation and driving factors to improve the accuracy of results. On this basis, the application of BBN not only effectively expresses the complex causal relationships among various variables in the process of grassland degradation, but also solves the problem of identifying key factors and assessing grassland degradation risks under uncertain conditions caused by a lack of information. The obtained result showed that the accuracies based on the confusion matrix of the slope of NDVI change (NDVIs), shrub-encroached grasslands, and grassland degradation indicators in the BBN model were 85.27, 88.99, and 74.37%, respectively. The areas under the curve based on the ROC curve of NDVIs, shrub-encroached grasslands, and grassland degradation were 75.39% (*P* < 0.05), 66.57% (*P* < 0.05), and 66.11% (*P* < 0.05), respectively. Therefore, this model could be used to infer the probability of grassland degradation risk. The results obtained using the model showed that the area with a higher probability of degradation (*P* > 30%) was 2.22 million ha (15.94%), with 1.742 million ha (78.46%) based on NDVIs and 0.478 million ha (21.54%) based on shrub-encroached grasslands. Moreover, the higher probability of grassland degradation risk was mainly distributed in regions with lower vegetation coverage, lower temperatures, less potential evapotranspiration, and higher soil sand content. Our research can provide guidance for decision-makers when formulating scientific measures for alpine grassland restoration.

## Introduction

Grasslands cover approximately 40% of the global area, and have significant effects on the production, daily life of humans, and ecology (Zong et al., 2021). However, these grasslands are currently degrading owing to the influence of human activity and climatic changes on grassland ecosystems. As a typical ecologically vulnerable area, alpine grasslands have significantly changed in terms of plant productivity and diversity, soil properties, and vegetation community. This has resulted in extensive degradation (Zhang et al., 2018; Miehe et al., 2019). Alpine grassland degradation has become an ecological and environmental issue that is of global concern (Seddon et al., 2016; Zhang et al., 2018). Take the Qinghai-Tibet Plateau as an example, whose coverage area of alpine grassland is 128.78 million ha (Liu et al., 2021). However, nearly 50% of the grasslands have been degraded owing to the interference of livestock husbandry and climatic changes (Teng et al., 2020). To relieve the ecological environmental pressure caused by alpine grassland degradation, the government of China (GOC) has implemented a plan to turn grazing land back into grassland and implement a policy of grassland ecological compensation. Although grassland vegetation is improving overall, intensive grassland degradation is occurring in local areas (Zhou et al., 2020; Wang Z. et al., 2021). Further, alpine grassland ecosystems are complex systems, making it difficult to solve all degradation problems through policy instruments alone. Hence, recognising and predicting grassland degradation patterns from the dual perspective of nature and humanity is imperative.

Previous studies on grassland degradation assessment are mainly based on remote sensing technology and parameter assessment systems (Gao et al., 2006; Feng et al., 2009; Lang et al., 2021). When using this method, indexes for grassland degradation are always uncertain, and most index parameters are set according to the subjective experience of researchers, resulting in the results being unreliable. Some scholars have detected grassland degradation using alarm signals (Lin et al., 2015; Han et al., 2018). However, this method had a small observation scope, and can only ascertain grassland degradation, without understanding its driving forces. In addition, previous studies do not take into consideration probability due to the complexity of grassland degradation mechanisms, the uncertainty of risk driving factors, and approaches limitations (Fleskens and Stringer, 2014; Farber, 2015). In fact, grassland degradation is influenced by multiple factors and the degradation trend should be a probability event. This risk assessment is determined by scientific cause and effect relationships where cause and effect are understood and predictable (although uncertainty is not quite zero) (French, 2015).

Therefore, an effective method for grassland degradation risk assessment – which can not only express the uncertainty of grassland degradation, but also understand the quantitative inference of grassland degradation risk probabilities according to the relationship between grassland degradation and driving factors – is required. As a method to measure the probability of risk occurrence, Bayesian belief networks (BBN) have been widely applied in accident risk assessment (Zywiec et al., 2021) and health risk prediction (Orak, 2020). Recently, the application of BBN to ecological environments is gradually increasing, including for decision support (Dang et al., 2019) and risk assessment (Plomaritis et al., 2018). BBN integrates several continuous variables (e.g., precipitation) and discrete variables (e.g., soil texture) through qualitative and quantitative analysis to construct an independent model (Zhu et al., 2020). Moreover, BBN inherits various data sources (e.g., expert knowledge, historical data, and empirical data) and transforms qualitative causality into a quantitative inference model based on probability calculations (Kerebel et al., 2019). The uncertainty of factors can be effectively solved because the uncertainty of the factor is transferred to the target variable through the conditional probability distribution table in the BBN model (Calder et al., 2019). Therefore, BBN not only can be used to assess the grassland degradation risk, but can also act as a decision support tool for stakeholders to make scientific measures.

The grassland of the Northwest Sichuan Plateau is located along the northeast edges of the Qinghai-Tibet Plateau, which is one of the five typical vulnerable grassland ecological regions in China. In this region, the grassland area is 13.93 million ha, accounting for more than 50% of the total area of the region, and has a high ecological value in terms of water conservation, soil and water conservation, and biodiversity protection. However, the ecosystem of local grasslands is becoming more and more vulnerable owing to the interference of humans and natural factors, which influences ecosystem services and the development of livestock husbandry (Li et al., 2020).

In this study, a quantitative grassland degradation risk assessment of the Northwest Sichuan Plateau was carried out by integrating the frequency ratio model (FR) model and BBN model. (1) The degradation pattern of grasslands in the study period was identified based on land use data and the normalized difference vegetation index (NDVI). (2) The driving risk factors of grassland degradation were discretized using the FR model and the BBN model structure was established according to knowledge regarding the grassland degradation and driving factors. (3) The importance of driving factors was identified based on a sensitivity analysis of the BBN model. (4) The probability of grassland degradation risk occurrence in each grid was predicted. On one hand, research conclusions can acquire objective and integrated grassland degradation states in the Northwest Sichuan Plateau. On the other hand, this study can assist decision-makers to formulate grassland restoration measures to maintain sustainable development in the region.

## Materials and Methods

### Study Area

**FIGURE 1:**
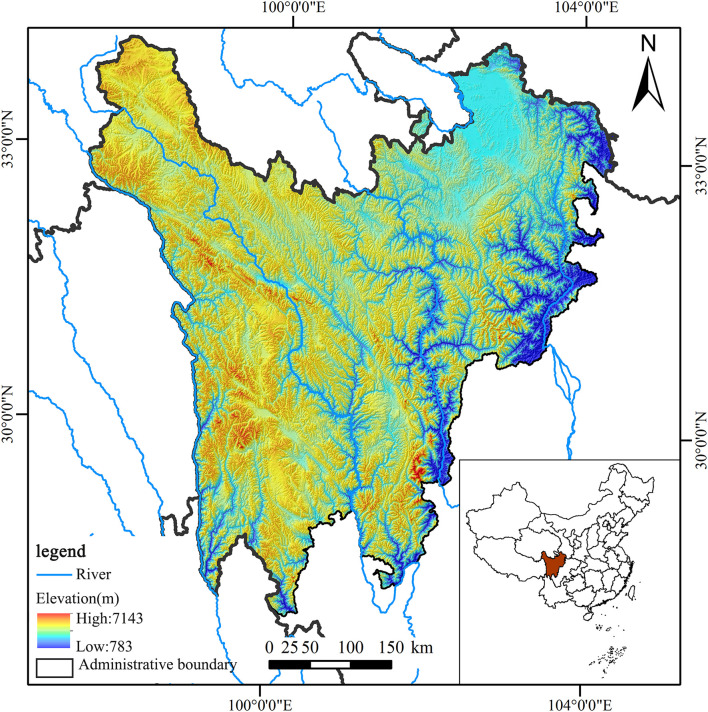
Location and elevation of the study area.

With a total area of approximately 13.93 million ha, the alpine grasslands of the Northwest Sichuan Plateau – located along the southeast edges of the Qinghai-Tibet Plateau (97°34′ – 104°43′E, 27°96′ – 34°31′N), is home to two prefecture-level cities (Ganzi Tibetan Autonomous Region and Aba Tibetan and Qiang Autonomous Prefecture) and 31 counties (districts) (Figure 1). In 2018, the population was about 2.14 million, which only accounted for 2.6% of the total population of the Sichuan Province. In the region, the terrain lifts gradually from the east to the west, and the elevation ranges between 783 and 7,143 m. Grasslands are the major land-use type in the Northwest Sichuan Plateau, which accounts for 59.92% of the total area. Moreover, a large area comprises an ecological reserve, which plays an important role in water conservation, water–soil conservation, and biodiversity protection in China.

Owing to the high sensitivity and vulnerability of alpine grasslands, ecological functions in local areas are degrading in response to human activities and climate change. In particular, there has been a significant increase in grassland degradation and insect attacks. The area of grassland degradation, desertification, and salinization has reached 9.39 km^2^, which accounts for 6.96% of the national value in China. Further, about 45% of the counties suffer from livestock overloading in the summer, which causes over-grazing in grasslands. Considering the ecological concerns and existing grassland degradation, the alpine grasslands of the Northwest Sichuan Plateau were chosen for grassland degradation risk assessment, which aims to help stakeholders recognize important spaces and allocate resources reasonably.

### Data Collection

In this study, eight datasets were used, as follows. (1) Land use and land cover (LULC) data in 2005 and 2018, with spatial resolution of 30 m, from the Resource and Environmental Science Data Center of the Chinese Academy of Sciences^1^; (2) Digital elevation model (Elevation) data from SRTM90m were provided by the Resource and Environmental Science Data Center of the Chinese Academy of Sciences (see text footnote 1); (3) Nighttime light data was published on the Harvard Dataverse^2^, with a spatial resolution of 500 m; (4) Geographic information data, including administrative boundaries, administrative centres, traffic network elements, and river basin datasets, were taken from the National Geomatics Center of China^3^; (5) Meteorological data from 2005 to 2018, including the precipitation, temperature, and potential evapotranspiration (ET_0_), were obtained from China’s meteorological data sharing service system^4^; (6) the MOD13Q1 NDVI from 2005 to 2018, with a spatial resolution of 250 m, was obtained from the United States Geological Survey^5^; (7) Soil texture data were taken from the 1:1,000,000 soil dataset of the National Tibetan Plateau Data Center^6^; and (8) the livestock numbers were provided by the local governmental department. In total, 18 potential factors affecting grassland degradation were selected (Table 1), and the spatial distribution of the indicators are shown in Supplementary Figure 1.

**TABLE 1:** Potential factors affecting grassland degradation.

Factors	Description	
Topographic	Elevation	Elevation (m)
	Slope	Slope (°)
Soil	Clay	Proportion of clay (%)
	Sand	Proportion of sand (%)
	Silt	Proportion of silt (%)
Climatic	Tm	Mean value of annual temperature (2005–2018) (°C)
	Ts	Slope of annual temperature change (2005–2018) (/)
	ETm	Mean value of annual potential evapotranspiration (2005–2018) (mm)
	ETs	Slope of annual potential evapotranspiration change (2005–2018) (/)
	Pm	Mean value of annual precipitation (2005–2018) (mm)
	Ps	Slope of annual precipitation change (2005–2018) (/)
Social and economic	Livestock	Mean value of the number of livestock (2005–2018) (cow unit)
	NTLm	Mean value of annual nighttime light (2005–2018) (/)
	NTLs	Slope of annual nighttime light change (2005–2018) (/)
	DW	Distance between each grid and its nearest water body (m)
	DR	Distance between each grid and its nearest road (m)
	DD/DC	Distance between each grid and its nearest administrative centre (m)
Others	NDVIm	Mean value of annual normalized difference vegetation index (2005–2018) (/)

### Methods

#### Concept of Bayesian Belief Networks

Bayesian belief networks is supported by a flexible network structure that can perform bottom-up reasoning or diagnostic analysis, along with top-down reasoning or predictive analysis (Rusek et al., 2021). They consist of nodes, which represent the random variables, and arrows, which represent the cause–effect relationship between variables. All variables in the BBN model are discretised into a limited number of states, and the causal relationship between a parent node X and a child node Y is quantified through a conditional probability table (CPT). For those nodes without a parent, the CPT transitions into a probability distribution with several possible states. These probabilities can be obtained through observational data, professional knowledge, or empirical observation (Kabir et al., 2015; Abebe et al., 2018). The primary advantage of using BBNs is that they reach probabilistic inferences or update their beliefs by integrating qualitative and quantitative data using the conditional probability theorem (Bicking et al., 2019) (Eq. 1). In the BBN model, the joint probability distribution (JPD) of related variables is obtained by multiplying the CPT of all nodes, as shown in Eq. 2. The JPD enables a BBN to effectively calculate the conditional probability of events based on the introduction of evidence variables. In the context of risk prediction, for example, the BBN can forecast the conditional probability of grassland degradation given information on evidence variables in each grid.


(1) $$\text{P}\,\left(\text{B}|\text{A}\right)=\frac{\text{P}\,\left(\text{A}|\text{B}\right)\times \text{P}\,\left(B\right)}{\text{P}\,\left(A\right)}$$

Equation 1 indicates that the posterior probability of an event *B* based on the data or evidence *A* is observed in terms of the prior probability of *B P*(*B*), the conditional probability of *A* given *B P*(*A*|*B*), and the prior or marginal probability of *A P*(*A*).


(2) $$\text{P}\left({\text{X}}_{1},{\text{X}}_{2},\ldots ,{\text{X}}_{n}\right)=\prod_{i=1}^{n}\text{P}\left({\text{X}}_{i}|\text{parent}\left({\text{X}}_{i}\right)\right)$$

Equation 2 is a formal representation of probability theory to calculate the JPD over a set of related variables.

**FIGURE 2:**
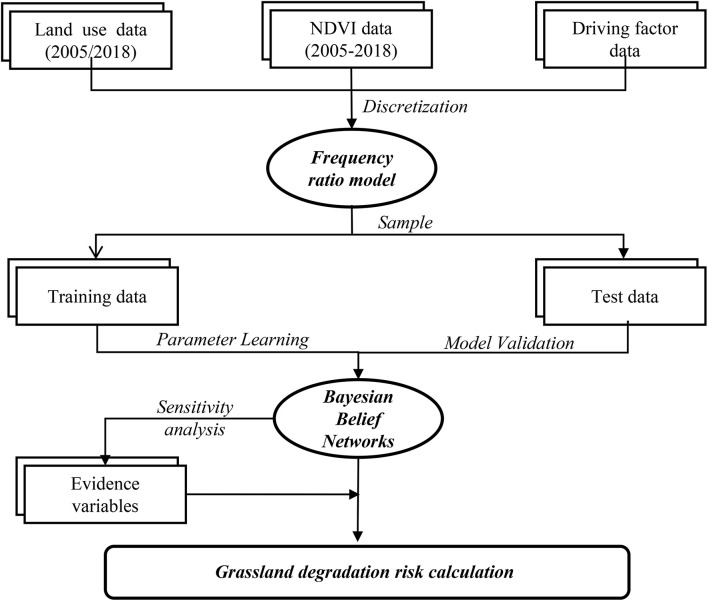
Design and application framework of the BBN model.

The construction of a BBN model includes model design and parameterization, model validation, and risk probability inference. The Genie and MATLAB software were used to construct the BBN model and infer the risk probability of grassland degradation. The design and application of the BBN model for the case study are presented in Figure 2.

#### Establish the Grassland Degradation Risk Inference Model

For macro-scale research, the characterization of grassland degradation based on NDVI is a commonly used method. NDVI is an important indicator of vegetation coverage, with which it has a significant linear relationship (Zhao et al., 2021). Therefore, for each grassland grid, the slope value of NDVI (NDVIs) from 2005 to 2018 could be used to indicate the trend of grassland change, and NDVIs values less than 0 could be regarded as grassland degradation. However, it is worth noting that NDVIs values lower than 0 do not indicate grassland degradation, because shrub-encroached grasslands in alpine regions are an important form of grassland degradation and do not necessarily lead to a reduction in NDVI (Fraser et al., 2014; May et al., 2020). Therefore, to more comprehensively identify the pattern of grassland degradation, we defined grassland degradation as non-encroached degraded grasslands with negative slope values of NDVIs (NDVIs < 0) and shrub-encroached grasslands.

**FIGURE 3:**
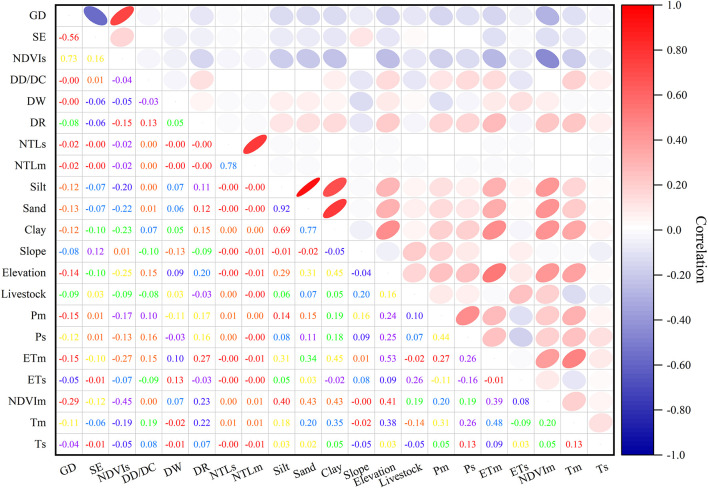
Correlation matrix between the selected variables for grassland degradation. GD, grassland degradation; NDVIs, slope of NDVI change; SE, shrub-encroached grasslands.

**FIGURE 4:**
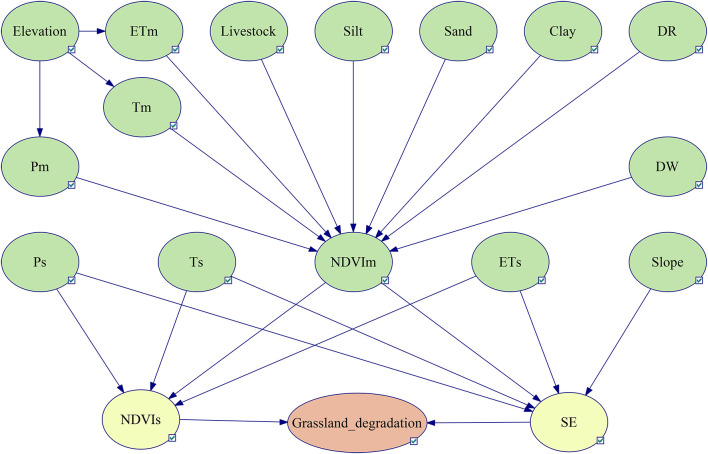
Bayesian conceptual network of grassland degradation risk inference. NDVIs, slope of NDVI change; SE, shrub-encroached grasslands.

Taking into account the non-stationarity of ecological processes, the diversity and incompleteness of data, and the complex relationships among factors, we selected 18 potential factors based on previous research results (Kane et al., 2017; Chen et al., 2020). The Pearson correlation coefficient was used to identify the correlation and intensity between potential factors and NDVIs or shrub-encroached grasslands. The results are shown in Figure 3, where the red pixels represent a positive correlation between two variables, blue pixels represent a negative correlation between two variables, and the flatter the ellipse, the larger the absolute value of the correlation coefficient. The results indicate that the selected potential factors had impacts on grassland degradation, other than NTLs, NTLm, and DD/DC. Therefore, NTLs, NTLm, and DD/DC were removed, because including them had no significant effect on the prediction results. In combination with the aforementioned analysis results, expert judgment, and historical data, the BBN-based grassland degradation risk model is constructed as shown in Figure 4.

#### Model Parameterization and Validation

First, the scientific division of driving factor states is the premise of the BBN model inference. As an efficient probabilistic method, the FR model can provide a more reliable prior knowledge for the BBN model of grassland degradation by calculating the frequency ratio of grassland degradation and impact factors in each interval. The larger the FR value, the higher the probability of grassland degradation (Yang et al., 2021). In this study, the FR not only characterizes the proportion of the area where grassland degradation occurs, but also explores the possibility of grassland degradation occurring (or not occurring)under given conditions. Thus, the intervals with similar frequency ratios can be merged to realize the scientific division of indicator factor status. It can be calculated as follows:


(3) $$\text{FR}=\frac{\text{a}/\text{b}\left(\%\right)}{\text{c}/\text{d}\left(\%\right)}$$

where *a* is the number of each factor’s grassland degradation, *b* is the number of total grassland degradation, *c* is the number of pixels in a given factor, and *d* is the total number of pixels in the study area.

Then, a case file was generated, which included 124,517 observations, with each row representing a sample. The case file was randomly divided into two partitions: a training set (*n* = 99,614; 80%) for model development, and a testing set (*n* = 24,903; 20%) for accuracy. Given the link structures, the training dataset was entered into the model as evidence to calculate the CPTs of each node in the BBN model.

Finally, to evaluate the accuracy of the BBN model predictions, a confusion matrix and receiver operating characteristic (ROC) curve were calculated using the testing set. The confusion matrix is a useful tool to ascertain the level of prediction accuracy by comparing the number of true values against the number of predicted values (Deng et al., 2016). The ROC curve can effectively measure the judgment ability of the model. If the model has good judgment ability, the ROC curve will be located above the diagonal of the coordinate axis, and the corresponding AUC will be greater than 0.5 (Anderson, 2019).

#### Grassland Degradation Risk Calculation

Through the evaluated BBN model, it is convenient to infer the probability of the target nodes in different combination states of evidence variables. The evidence variables with a significant contribution to grassland degradation were chosen through sensitivity analysis. Variance of belief (VB) – based on variance reduction – and mutual information (MI) – based on entropy reduction – are often used as sensitivity analysis indicators to quantitatively evaluate whether network nodes sensitively perceive changes in other nodes (Shi et al., 2020). Therefore, this study used the VB and MI to assess the sensitivity of input variables relative to the target variables. VB and MI are calculated as follows:


(4) $$\text{VB}=\text{V}\,\left(S\right)-\text{V}\,\left(\text{S}|\text{I}\right)=\,\sum_{s}\text{P}\,\left(s\right)\times {\left(\text{s}-\text{E}\,\left(S\right)\right)}^{2}-\sum_{s}\text{P}\,\left(\text{s}|\text{I}\right)\times {\left(\text{s}-\text{E}\,\left(\text{S}|\text{I}\right)\right)}^{2}$$

(5) $$\text{MI}=H\,\left(S\right)-\text{H}\,\left(\text{S}|\text{I}\right)=\sum_{s}\sum_{i}\text{P}\,\left(s,i\right)\,l\,o\,g_{2}\,\left(\frac{\text{P}\,\left(s,i\right)}{\text{P}\,\left(s\right)\times \text{P}\,\left(i\right)}\right)$$

where *S* is the target variable, *I* is another variable, and *s* and *i* represent the states of *S* and *I*, respectively. The larger the value of *VB* and *MI*, the stronger influence of the driving factor on the target variable.

According to the results of the sensitivity analysis, we selected six driving factors with the highest sensitivity to target variable changes as the evidence variables. The CPT and probability distribution of each node in the BBN model can infer the CPT of the target variable under given conditions (Hao et al., 2018). Therefore, by integrating the evidence variables of each grid into the established BBN model, we can obtain the potential grassland degradation risk in the grid.

## Results

### Grassland Degradation Pattern

**FIGURE 5:**
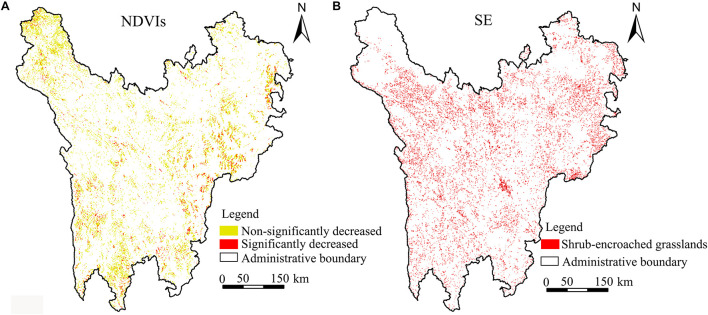
Grassland degradation from 2005 to 2018. **(A)** NDVIs, slope of NDVI change. **(B)** SE, shrub-encroached grasslands.

The dynamic variation trend of NDVI in each grid from 2005 to 2018 was calculated (Figure 5A). According to statistical results, the area of NDVIs less than 0 was 2.433 million ha, with 11.39% attributed to significantly decreased areas and 88.61% to non-significantly increased areas. In view of the spatial distribution, these areas are mainly distributed in Shiqu County, Songpan County, Daocheng County, and the junction of Maerkang County, Li County, and Xiaojin County. Based on land use data (Supplementary Figure 2) in 2005 and 2018, shrub-encroached grasslands were recognized, as shown in Figure 5B. The results showed that shrub-encroached grasslands covered an area of 1.689 million ha, and the areas of the degraded grids were larger in Daofu County, Songpan County, and Wenchuan County. Overall, the grassland degradation appeared in scattered areas and in a wide range. According to the results of the overlay analysis of shrub-encroached grasslands and NDVIs, a spatially intersecting area of 1.357 million ha was identified. This meant that the 1.553 million ha of degraded grasslands had been identified through shrub-encroached grasslands, which offset the shortcomings of grassland degradation recognition based on NDVIs only.

#### Driving Factors Analysis

**FIGURE 6:**
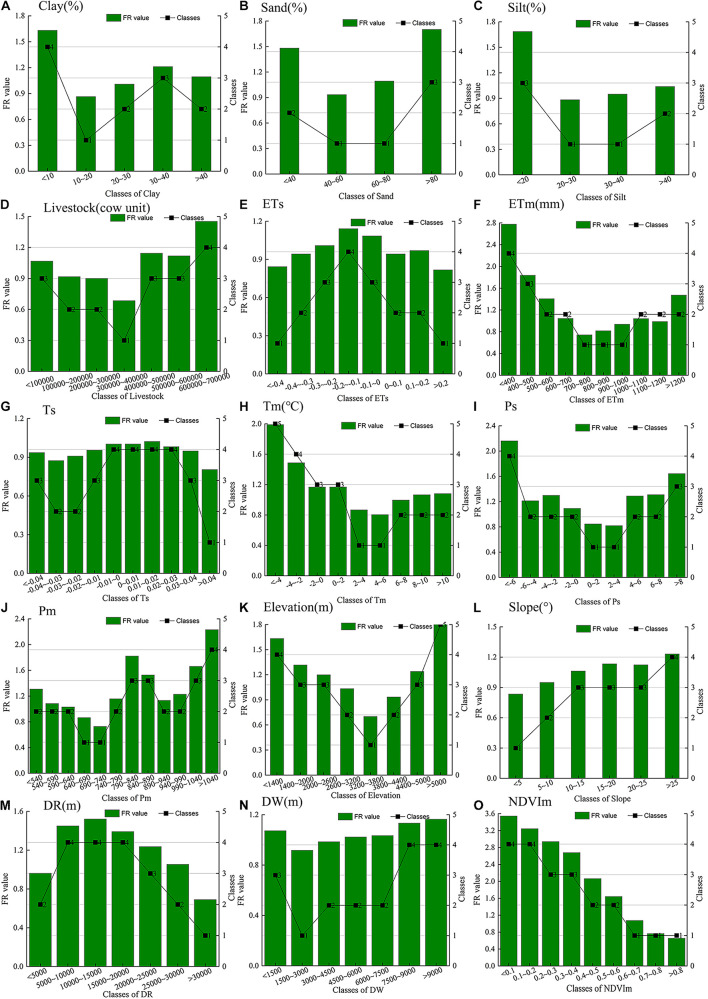
Relationship between the grass degradation and the conditioning factors. **(A)** Clay. Proportion of clay. **(B)** Sand (%). Proportion of sand. **(C)** Silt (%). Proportion of silt. **(D)** Lm (cow unit). Mean value of the number of livestock. **(E)** Ts. Slope of annual temperature change. **(F)** Tm (°C). Mean value of annual temperature. **(G)** ETs. Slope of annual potential evapotranspiration change. **(H)** ETm (mm). Mean value of annual potential evapotranspiration. **(I)** Ps. Slope of annual precipitation. **(J)** Pm (mm). Mean value of annual precipitation. **(K)** DEM (m). Elevation. **(L)** Slope (°). Slope. **(M)** DR (m). The distance between each grid and its nearest road. **(N)** DW (°). The distance between each grid and its nearest water body. **(O)** NDVIm. Mean value of annual normalized difference vegetation index.

The FR model was used to identify the relationship between screened driving factors and grassland degradation, and then the driving factors were discretized based on similar frequency ratios (Figure 6). For the soil silt content, the class with a soil silt content of less than 10% had the highest FR value, indicating that grassland degradation has the highest probability of occurrence. The results of soil sand content and soil silt content showed that most grassland degradation occurred at values >80% and <10%, respectively. The livestock results indicated that the >600,000 class had a higher FR value than the other classes, which indicated a high probability of grassland degradation. For Tm and Ts, the classes of −0.01 −0.03 and <−4°C, respectively, had a higher grassland degradation occurrence. The highest FR value of ETs, ETm, Ps, and Pm belonged to the −0.2−0.1 class, <400 mm, <−6, and >1,040 mm, respectively. Interestingly, some factors exhibited obvious spatial variations in terms of the FR results. For example, the FR results for the >5,000 m class of elevation and the >25° class of slope were greater than those of the other classes, indicating that the grassland degradation occurred close to high altitudes and high slopes. Regarding the DR and DW factors, grassland degradation occurred more easily in areas 5,000–20,000 m away from roads and areas more than 7,500 m away from rivers. The FR value of NDVIm decreased with increases in vegetation coverage, which was not only consistent with prior knowledge of grassland degradation, but also verified the reliability of the experimental dataset. Based on the above analysis, the screened driving factors were classified according to the FR value. The results of the aforementioned classes can be found in Supplementary Table 1.

#### Parameter Learning and Model Validation

**FIGURE 7:**
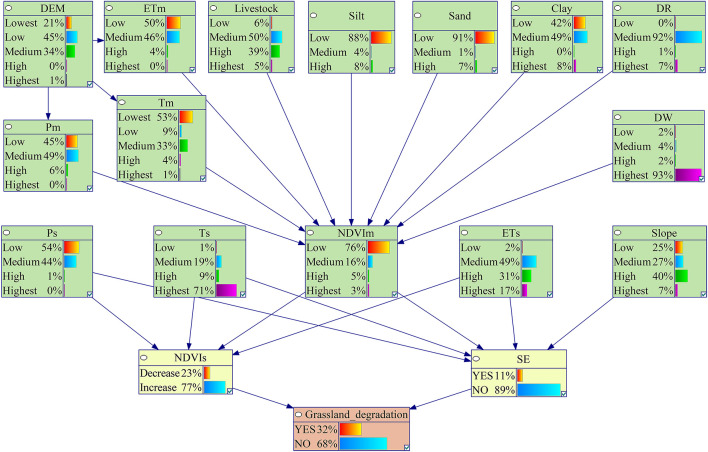
Results of parameter learning in the BBN model. NDVIs, slope of NDVI change; SE, shrub-encroached grasslands.

Based on the above discretization results regarding variables related to grassland degradation, 80% of the training set was selected randomly for the parameterization of the BBN model (Figure 7). According to the parameterization results, 31.58% of grassland grids were experiencing degradation trends, indicating that the severity of grassland degradation in the study area requires the attention of stakeholders. Furthermore, in comparison to the grid number of shrub-encroached grasslands (10.58%), the grid number of non-encroached degraded grasslands (NDVI <0) caused by human activities and climatic changes is higher (22.51%).

**FIGURE 8:**
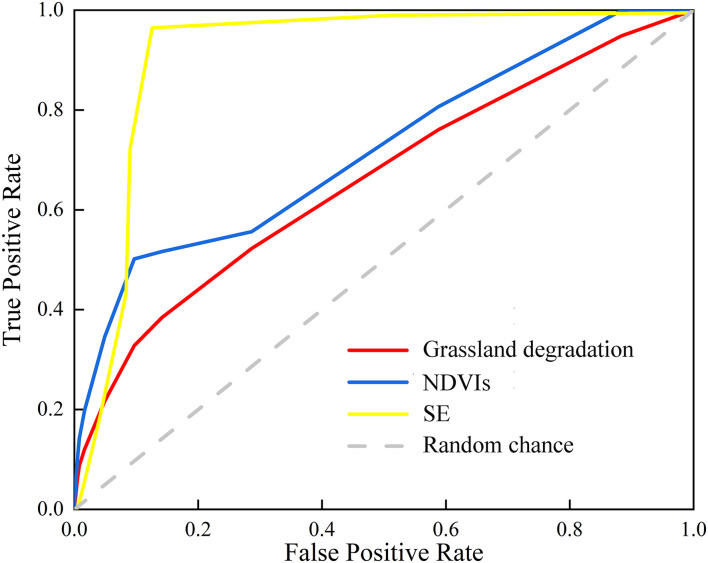
ROC curve of NDVIs, shrub-encroached grasslands, and grassland degradation. NDVIs, slope of NDVI change; SE, shrub-encroached grasslands.

The NDVIs, shrub-encroached grasslands, and grassland degradation were predicted by entering the test set into the BBN model. The precision of the BBN model was evaluated based on the confusion matrix and ROC curve. The results of the confusion matrix showed that the prediction accuracy of NDVIs, shrub-encroached grasslands, and grassland degradation was 85.27, 88.99, and 74.37%, respectively. The AUCs based on the ROC curve of NDVIs, shrub-encroached grasslands, and grassland degradation were 75.39% (*P* < 0.05), 66.57% (*P* < 0.05), and 66.11% (*P* < 0.05), respectively (Figure 8). This made it clear that the model accuracy met the criterion for grassland degradation risk assessment.

#### Sensitivity Analysis of the Target Variables

**FIGURE 9:**
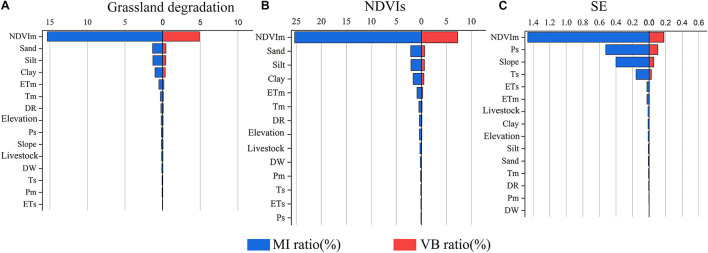
Sensitivity analysis of all driving factors. **(A)** Grassland degradation. **(B)** NDVIs, slope of NDVI change. **(C)** SE, shrub-encroached grasslands.

Nodes of NDVIs, shrub-encroached grasslands, and grassland degradation were chosen as target variables for sensitivity analysis. Figure 9 showed that the ratio of VB and MI exhibited a linear relationship; the higher value of MI, the higher value of VB. A high value of the ratio of VB and MI indicates the significant influence of the node on the target node. With respect to the nodes of grassland degradation and NDVIs, the variables of NDVIm, sand, silt, clay, ETm, and Tm had a high sensitivity (VB > 0.1%), indicating that they made significant contributions to grassland degradation. Therefore, significant attention should be paid to controlling the vegetation coverage, soil texture, and climatic factors in the grassland degradation regions in the future. For nodes of shrub-encroached grasslands, the higher sensitivity of NDVIm (VB = 0.179%) and Ps (VB = 0.106%) indicated that vegetation coverage and precipitation have significant influences on grassland degradation.

**FIGURE 10:**
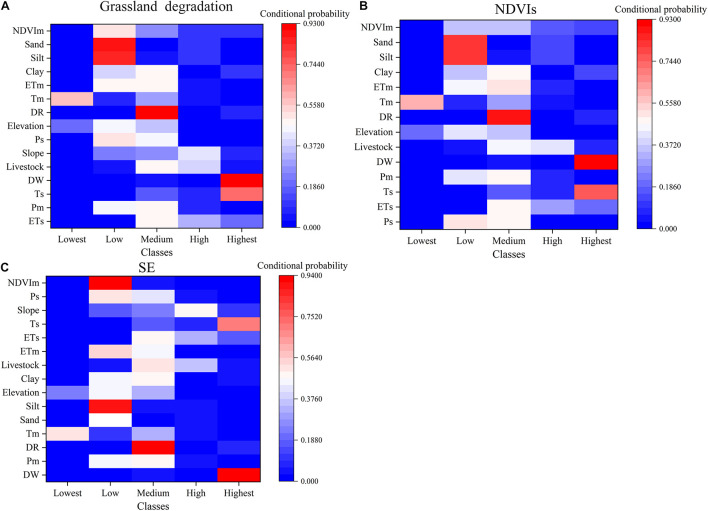
Heat maps showing the conditional probabilities driving NDVIs, SE, and grassland degradation. **(A)** Grassland degradation. **(B)** NDVIs, slope of NDVI change. **(C)** SE, shrub-encroached grasslands.

Heat maps showed the probability relationship between the state of the target variables and the influencing factors (Figure 10). The reddest pixels indicated that the factor has the highest conditional probability under the states of the given target variables. The bluest pixels indicated that the factor has the lowest conditional probability under the state of the given target variables. For example, the optimal combination of factors that had significant influences on grassland degradation was as follows: {NDVIm = Low, sand = Low, silt = Low, clay = Medium, ETm = Medium, Tm = Lowest}. For NDVIs, the probability of grassland degradation was relatively high when the optimal combination of factors was as follows: {NDVIm = Medium, sand = Low, silt = Low, clay = Medium, ETm = Medium, Tm = Lowest}. The probability of shrub-encroached grasslands in grids was relatively high when the grid was {NDVIm = Low, Ps = Low, slope = High, Ts = Highest, ETs = Medium, ETm = Low}. Therefore, attention should be paid to regions in the factor state combination that met the above conditions in the next stage.

#### Grassland Degradation Risk Probability Calculation

Based on the sensitivity analysis results (Figure 9) and corresponding principles, the evidence variables for NDVIs, shrub-encroached grasslands, and grassland degradation were chosen to infer the probability of the land degradation risks for each grid unit. These were NDVIm, sand, silt, clay, ETm, and Tm in NDVIs index and grassland degradation index. In the shrub-encroached grasslands index, these variables were NDVIm, Ps, slope, Ts, ETs, and ETm.

**FIGURE 11:**
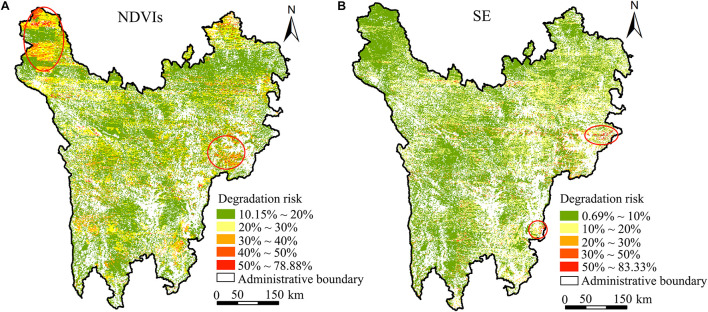
Inference probability of grassland degradation risks based on NDVIs and SE. **(A)** NDVIs, slope of NDVI change. **(B)** SE, shrub-encroached grasslands.

Figure 11 showed the probability of the degradation risk identified by NDVIs and shrub-encroached grasslands indicators in each grid according to the selected evidence variables. The results showed that the probability ranges of the degradation risk of NDVIs and shrub-encroached grasslands in the study area were 10.15–78.88% and 0.69–83.33%, respectively. According to grading results of the risks, the area of high-risk grassland degradation (*P* > 30%) predicted by NDVIs is 1.742 million ha, which accounted for 12.51% of the total study area. Moreover, these regions were primarily distributed in Shiqu County and the junction areas of Li County, Xiaojin County, and Wenchuan County, corresponding to areas with the “low” state of NDVIm, sand, and silt; the “medium” state of clay and ETm; and the “lowest” state of Tm. The area of high-risk grassland degradation (*P* > 30%) predicted by shrub-encroached grasslands is 0.636 million ha, which accounted for 4.57% of the total area, and these regions were mainly distributed in Mao County and Luding County, corresponding to areas with a “low” state of NDVIm, Ps, and ETm; “high” state of slope; “highest” state of Ts; and “medium” state of ETs, as expected.

**FIGURE 12:**
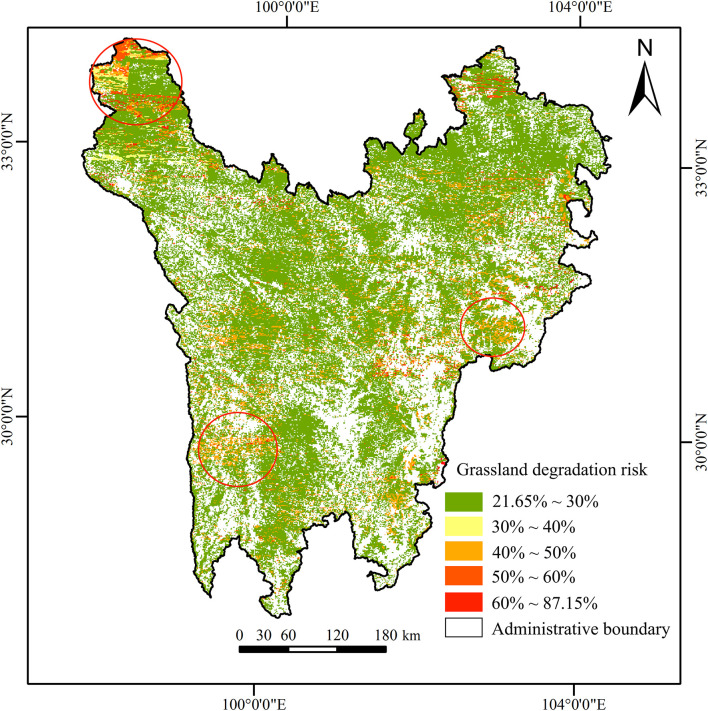
Distribution of grassland degradation risk.

The result of grassland degradation risk in each grid was predicted by the selected evidence variables, as shown in Figure 12. The risk probability values were about 21.65–87.15%. The region of high-risk grassland degradation (*P* > 30%) covered 2.22 million ha, accounting for 15.94% of the total study area. By comparing the high-risk degradation probabilities identified by NDVIs and shrub-encroached grasslands indicators in grids, it was found that the degradation area based on NDVIs was 1.742 million ha (78.46%) and the degradation area based on shrub-encroached grasslands was 0.478 million ha (21.54%). In terms of spatial distribution, regions with high-risk grassland degradation were quite scattered; however, they were clustered in local areas (e.g., the junction area of Shiqu County, Batang County, and Litang County, and the junction area of Li County, Wenchuan County, and Xiaojin County). It can be seen from Table 2 and Supplementary Table 1 that the grassland degradation mainly occurred in regions with lower vegetation coverage, higher soil sand content, lower soil clay and silt content, lower temperature, as well as less potential evapotranspiration, indicating that natural factors had stronger influences on vegetation degradation than human factors. Moreover, although this study chose 30% as the threshold for recognising high grassland degradation risks, there is uncertainty in terms of grassland degradation owing to the inherent nature of probability. As a result, regions with grassland degradation risk probabilities lower than 30% could have degraded, and regions with probabilities higher than 30% could have experienced no degradation. For example, grids with the highest degradation probability (87.15%) could also have no degradation.

**TABLE 2:** Comparison of evidence variables in different states in high-risk areas.

Indicator	Classes of evidence variable				
	
	Lowest	Low	Medium	High	Highest
NDVIm	–	49.8%	36.3%	11.8%	2.1%
Sand	–	41.1%	4.3%	54.6%	–
Silt	–	39.4%	4.3%	56.3%	–
Clay	–	0.9%	30.4%	0.1%	68.6%
ETm	–	0.2%	47.9%	51.8%	0.1%
Tm	32.9%	0.2%	48.4%	8.4%	10.1%

## Discussion

In this study, alpine grassland degradation in Northwest Sichuan Plateau was assessed by integrating the FR model and the BBN model. First, the driving factors of grassland degradation were discretized using the FR model. Then, a BBN model was established to identify the driving factors of grassland degradation and quantitatively evaluate the probability of degradation risks.

For the grassland degradation analysis, NDVIs and shrub-encroached grasslands were chosen as the grassland degradation index. In previous studies, most alpine grassland degradation on the macro scale was based on NDVIs, and the effects of shrub-encroached grasslands in alpine regions were ignored (which are one of the most important forms of alpine grassland degradation), resulting in missing degradation grids (Chen et al., 2020). To offset the limitations of recognition based on NDVIs only, grassland degradation was identified herein by combining shrub-encroached grasslands and the slope of NDVI change, which could realize the complete characterization of macro-scaled grassland degradation, and is conducive to understanding the states of different dominant degradation types. Furthermore, states of driving factors of NDVIs and shrub-encroached grasslands were divided by calculating the frequency ratios of different intervals of driving factors and combining intervals with similar frequency ratios (De Santana et al., 2021). The FR method obtains a prior probability, and then the BBN model is used to infer the posterior probability of the event. Compared with the node classification method based on the characteristics of the node itself in the previous studies (Dai et al., 2021; Sakib et al., 2021), the FR method is graded the driving factors based on the importance of each attribute interval of the factor to the susceptibility of the event, which is more scientific. Therefore, as the premise of the BBN model interference, the FR model can provide a relatively reliable prior probability.

In the process of grassland degradation risk assessment, the model structure and parameterization are the key steps of BBN modelling. In this study, the grassland degradation risk assessment model was constructed by integrating correlation analysis, expert experience, and previous research conclusions, which were effectively used to determine the complex causal relationships among variables during grassland degradation (Carriger and Parker, 2021). In comparison to previous grassland degradation risk assessment methods, the BBN-based grassland degradation risk assessment model was based on the relationship between grassland degradation and driving forces, which could deduce the uncertainty of grassland degradation that was caused by insufficient and incomplete relevant information or knowledge (Luu et al., 2009). Furthermore, the model could be applied to reassess the grassland degradation risk when new information or data from nodes were updated or replaced, helping decision-makers formulate appropriate management measures (Dai et al., 2021). Hence, the BBN method showed better reliability and practicability. According to the results of grassland degradation risk probability in the BBN model, the potential degradation risk in most regions in the study area was maintained at a low level (21.65–30%). However, local regions still had relatively high degradation risk probabilities, which primarily include the grassland restoration regions that decision-makers need to focus on in the future. In terms of the probability of degradation risks, it must be noted that the degradation risk in the BBN model is a concept of probability, which means that it is inherently uncertain (Rohmer, 2020). In other words, places with the lowest degradation probability (21.65%) could also experience degradation, and those with the highest degradation probability (87.15%) could experience no degradation. For this reason, the Bayesian results must be understood in terms of probability.

The sensitivity analysis results of target variables help us recognize the influence of factors on grassland degradation, facilitating the formulation of scientific and effective ecological restoration measures (Hao et al., 2018). The results showed that the vegetation coverage, soil texture, and climatic factors of grids influence grassland degradation significantly, while terrain and human activity-related factors had relatively smaller effects in the study area. This could be because of the low levels of human activity due to a small population in the study area; this caused grassland degradation to be mainly affected by natural factors (Gang et al., 2014; Wang et al., 2017). However, it is worth noting that the IPCC report clearly stated that human activities significantly affect climate change, so that grass degradation related to climate change is indirectly affected by human activities (Intergovernmental Panel on Climate Change, 2021). In addition, the probability correlations between grassland degradation and important driving factors showed that the highest probability of grassland degradation occurred when the state combination of driving factors met the following conditions: {NDVIm = Low, sand = Low, silt = Low, clay = Medium, ETm = Medium, Tm = Lowest}. This was consistent with geographic features in high-risk regions predicted by the BBN model. Therefore, the grassland restoration measures in the future should pay keen attention to regions with low vegetation coverage, high soil sand content, less potential evapotranspiration, and low temperatures. Moreover, the probability of grassland degradation was relatively low in regions with high vegetation coverage, high soil clay content, high temperature, and high potential evapotranspiration. Based on previous studies, there is evidence that human activities can improve the status of the above factors to a certain extent, such as the Returning Grazing Land to Grassland Project can increase the vegetation coverage (Shao et al., 2016); fertilization or soil amendments can improve soil properties (Wang X. et al., 2021). The findings from this study provide a reference basis for the restoration of grassland ecosystems.

Overall, the BBN method used in this study is most suitable in the situation characterised by large uncertainties, long time frames, and the influence of socio-economic and biophysical changes. However, in addition to the impact of the driving factor status, the temporal and spatial scale along with the data update rate will also affect the accuracy of grassland degradation risk assessment. To improve the accuracy of the assessment and the reliability of grassland ecosystem restoration decisions, on the basis of ensuring the timeliness and accuracy of the data, other potential influencing factors should also be identified; this should be followed by the optimization of the BBN model should improve the accuracy of grassland degradation risk inferences.

## Conclusion

In this study, we use the alpine grassland of Northwest Sichuan Plateau as an example to predict the grassland degradation risk probability. We also propose suggestions for the scientific and reasonable restoration of grassland ecosystems, elucidating the advantages of using this method. The results regarding the grassland degradation pattern indicated that the area of grassland degradation characterized by NDVIs is 2.433 million ha, with 11.39% in significantly decreased areas and 88.61% in non-significantly increased areas. The grassland degradation area characterized by shrub-encroached grasslands was 1.6886 million ha. Moreover, overlapping results of shrub-encroached grasslands and NDVIs less than 0 indicated a spatial intersection of 1.357 million ha. In other words, a total of 1.553 million ha of grassland degradation was recognized through shrub-encroached grasslands. In the BBN model, the prediction accuracy based on the confusion matrix of NDVIs, shrub-encroached grasslands, and grassland degradation was 85.27, 88.99, and 74.37%, respectively. The area under the curves based on the ROC curve of NDVIs, shrub-encroached grasslands, and grassland degradation were 75.39% (*P* < 0.05), 66.57% (*P* < 0.05), and 66.11% (*P* < 0.05), respectively. This proved that the proposed BBN model met the requirements and could be used to predict the grassland degradation risk probability. According to model inference results, the area of high degradation risk probability (*P* > 30%) was 2.22 million ha (15.94%), with 1.742 million ha (78.46%) characterized by NDVIs and 0.478 ha (21.54%) characterized by shrub-encroached grasslands. Furthermore, the sensitivity analysis indicated that grassland degradation had a relatively high sensitivity to NDVIm, sand, silt, clay, ETm, and Tm, and regions with low vegetation coverage, high soil sand content, less evapotranspiration and low potential temperature could easily experience grassland degradation. Such regions included the junction area of Shiqu County, Batang County, and Litang County, and the junction area of Li County, Wenchuan County, and Xiaojin County.

Identifying and quantifying the complex relationships between driving factors and grassland degradation is crucial for grassland degradation assessment; the BBN method not only provides an effective solution to solve this problem, but assists stakeholders in formulating scientific decisions on grassland ecosystem restoration. Therefore, the BBN model could be used as a decision-support instrument for restoring grassland ecosystems, along with maintaining regional ecological safety and sustainable development.

## Data Availability Statement

The datasets presented in this study can be found in online repositories. The names of the repository/repositories and accession number(s) can be found in the article/Supplementary Material.

## Author Contributions

SZ and LP: conceive the ideas. SZ: methodology, investigation, formal analysis, software, data curation, visualization, and writing – original draft. LP: methodology, formal analysis, project administration, supervision, writing, review, and editing. Both authors read, commented on, and approved this version of the manuscript.

## Conflict of Interest

The authors declare that the research was conducted in the absence of any commercial or financial relationships that could be construed as a potential conflict of interest.

## Publisher’s Note

All claims expressed in this article are solely those of the authors and do not necessarily represent those of their affiliated organizations, or those of the publisher, the editors and the reviewers. Any product that may be evaluated in this article, or claim that may be made by its manufacturer, is not guaranteed or endorsed by the publisher.

## Footnotes

1
http://www.resdc.cn/

2
https://doi.org/10.7910/DVN/YGIVCD

3
https://www.openstreetmap.org/

4
http://data.cma.cn/

5
https://www.usgs.gov/

6
http://data.tpdc.ac.cn/
