# Supplementary material for: Applying Bayesian Belief Networks to Assess Alpine Grassland Degradation Risks: A Case Study in Northwest Sichuan, China
Source: Front Plant Sci. 2021 Nov 4;12:773759. doi: 10.3389/fpls.2021.773759 (PMC8600186; doi:10.3389/fpls.2021.773759)
Supplement: Supplementary file 2 [file Data_Sheet_2.docx]

Supplementary Material

# Supplementary Material 1

Grassland degradation is usually affected by biophysical and socioeconomic factors(Liu et al., 2019; Zhou et al., 2017). Based on objective data and scientific knowledge(Chen et al., 2020; Eddy et al., 2017), 18 potential factors affecting grassland degradation had been initially selected. According to previous research (Sha et al., 2016), temperature, precipitation and potential evapotranspiration factors were selected to characterize climate indicators, and the mean value and trend of them were used as climate variables. Topographic data and soil characteristics were respectively characterized by elevation, slope, and soil texture (sand, clay, and silt soil content). Socioeconomic factors were expressed by the number of livestock, night light index, and the distance between the center of each grassland grid and its nearest water body, roads and county town. According to relevant standards (Ministry of Agriculture and Rural Affairs of the People’s Republic of China, 2015), the number of livestock was transformed into the cattle units using the formula below. In addition, the mean value of NDVI was also considered as an influencing factor of grassland degradation, as it had the highest Pearson Correlation Coefficient with shrub-encroached grasslands and the slope value of NDVI, compared with other potential factors.

$$X_{total} =X_{c} +0.8\times X_{h} +X_{m} +0.6\times X_{d} +0.2\times X_{s} +0.025\times X_{r}$$

where $X_{total}$was the total number of cow unit; $X_{c}$was the total number of cows; $X_{h}$was the total number of horses; $X_{m}$was the total number of mules;$X_{d}$was the total number of donkeys; $X_{s}$was the total number of sheep;$X_{r}$was the total number of rabbits.


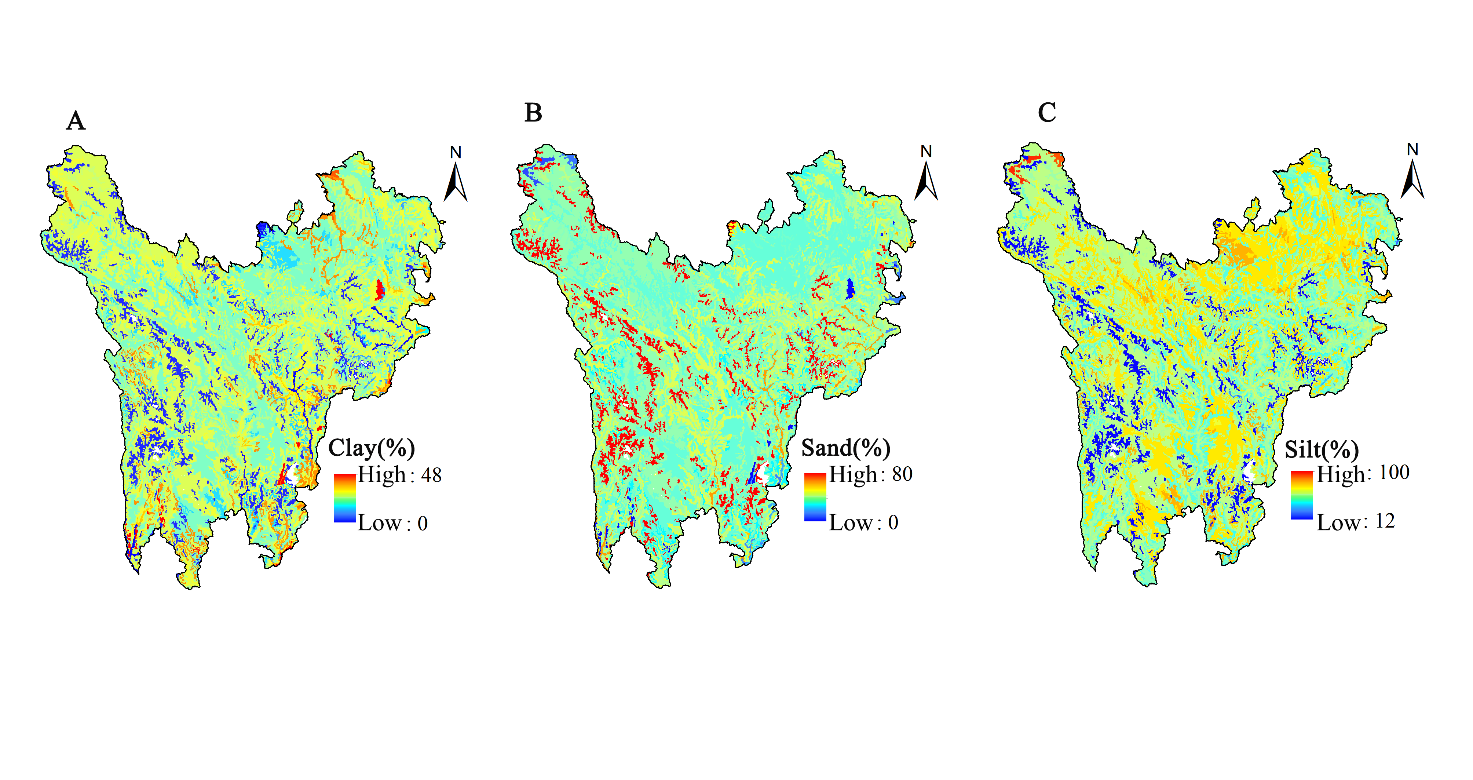


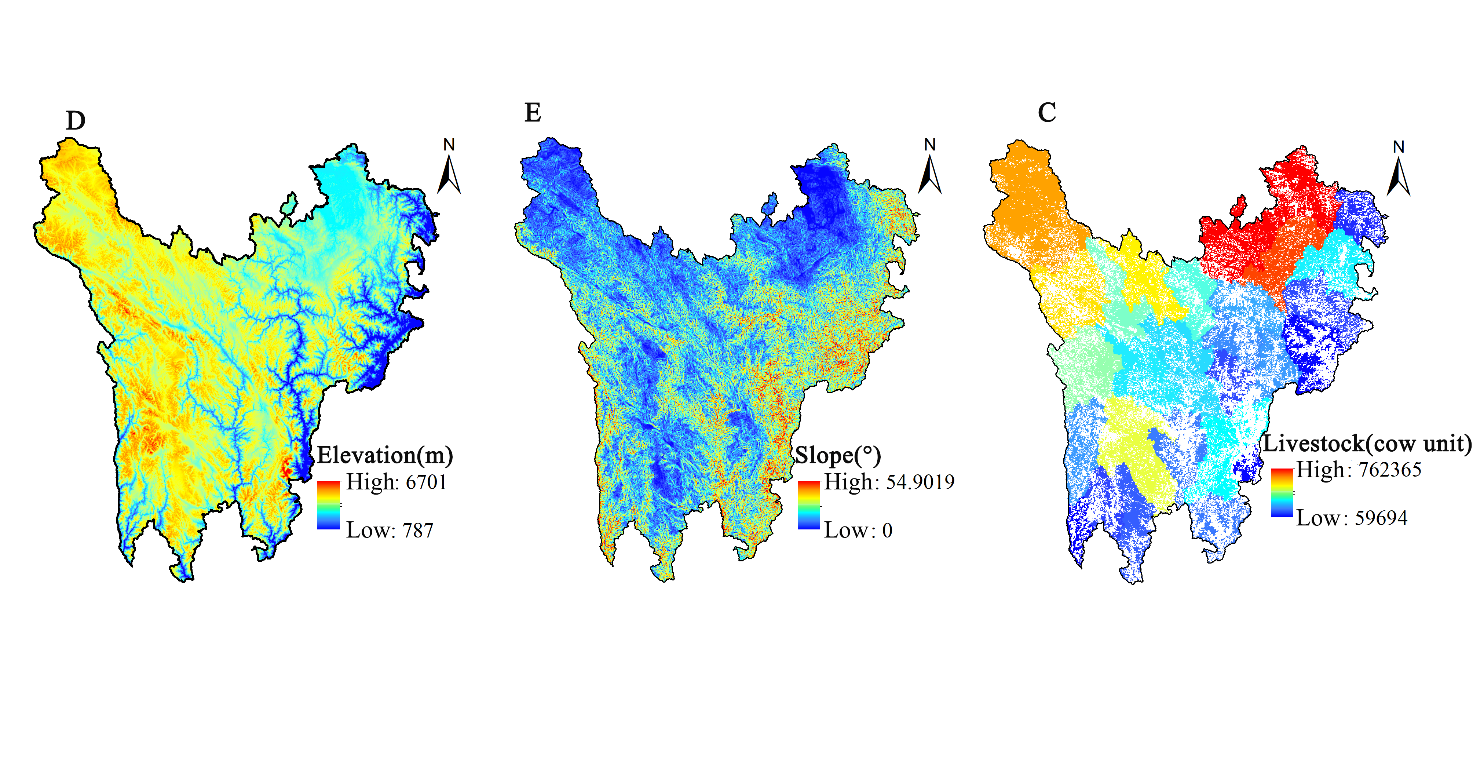

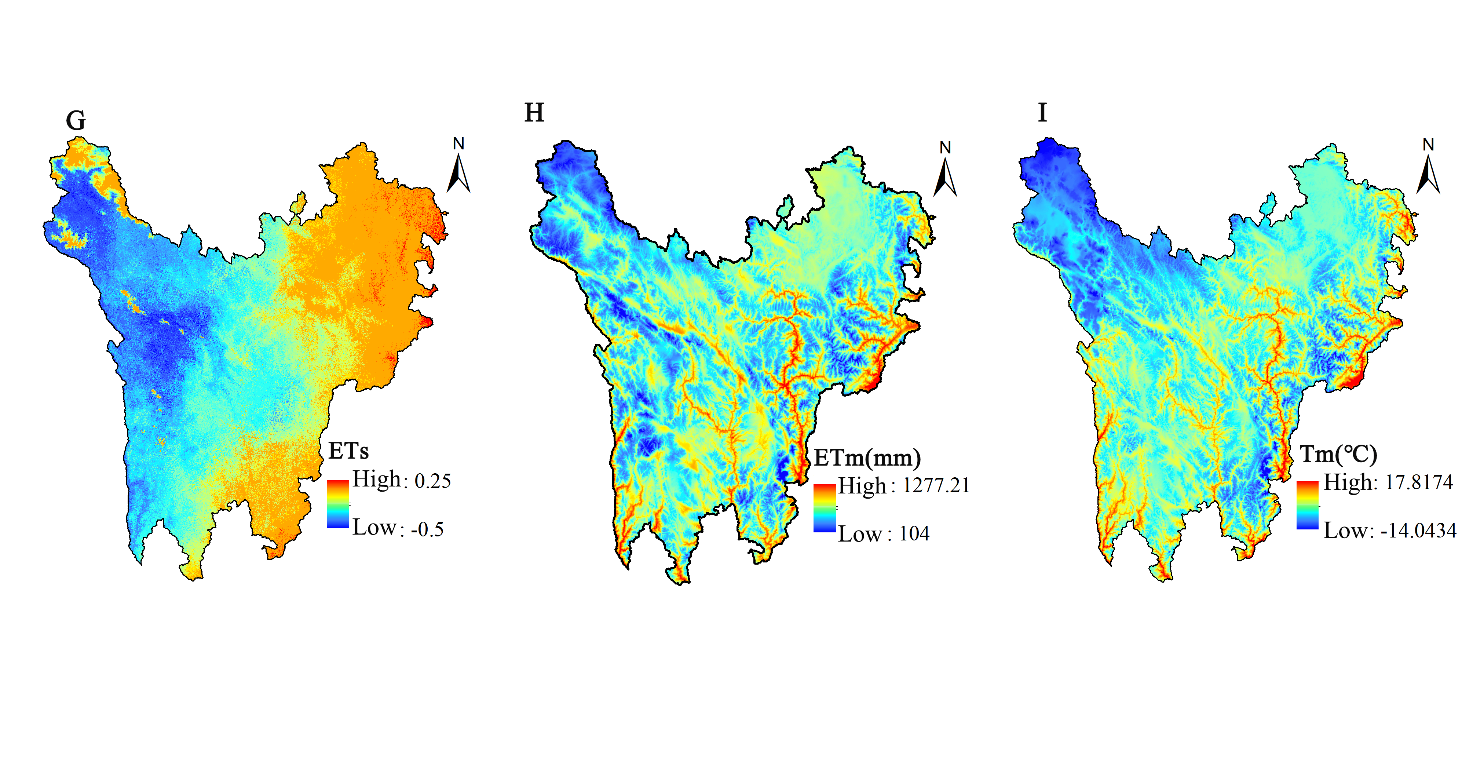

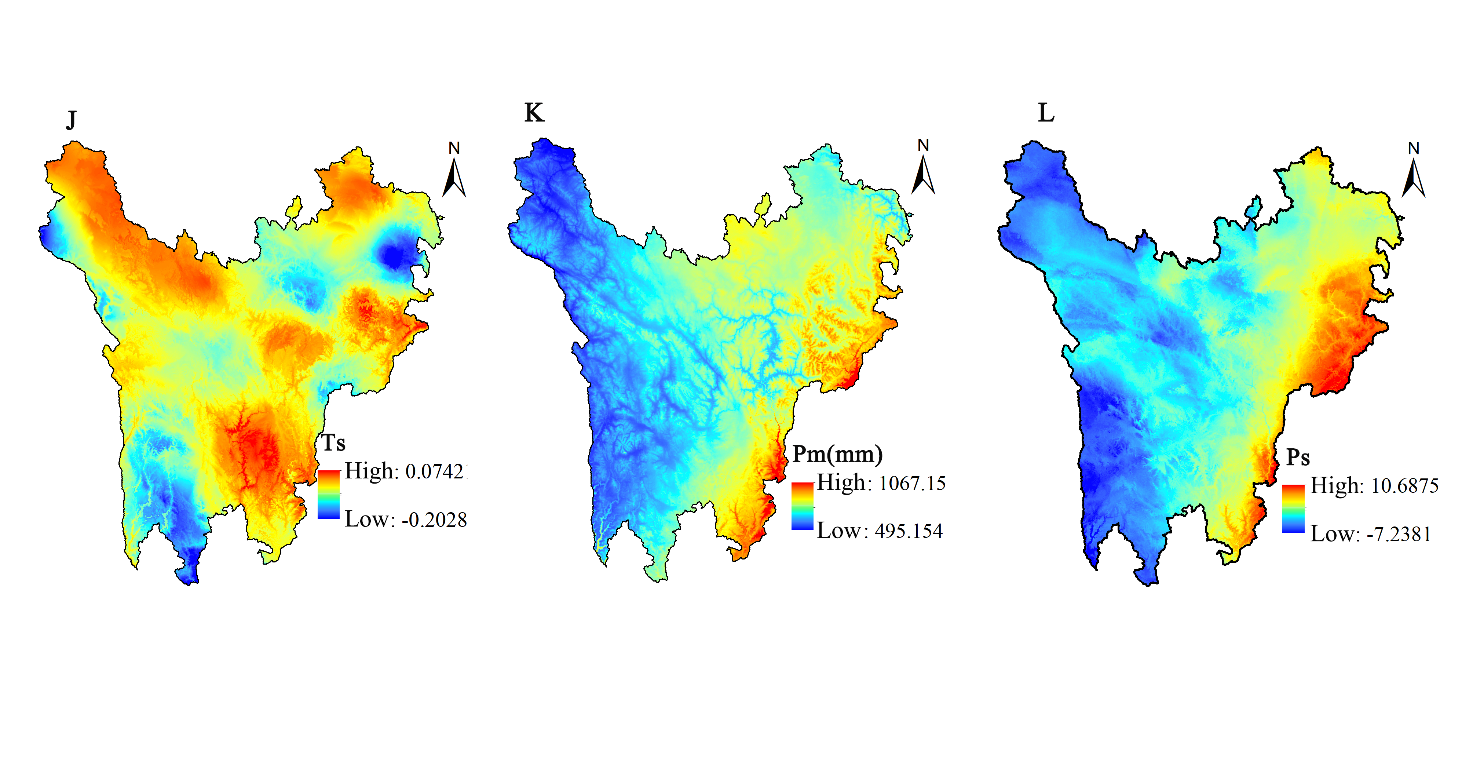

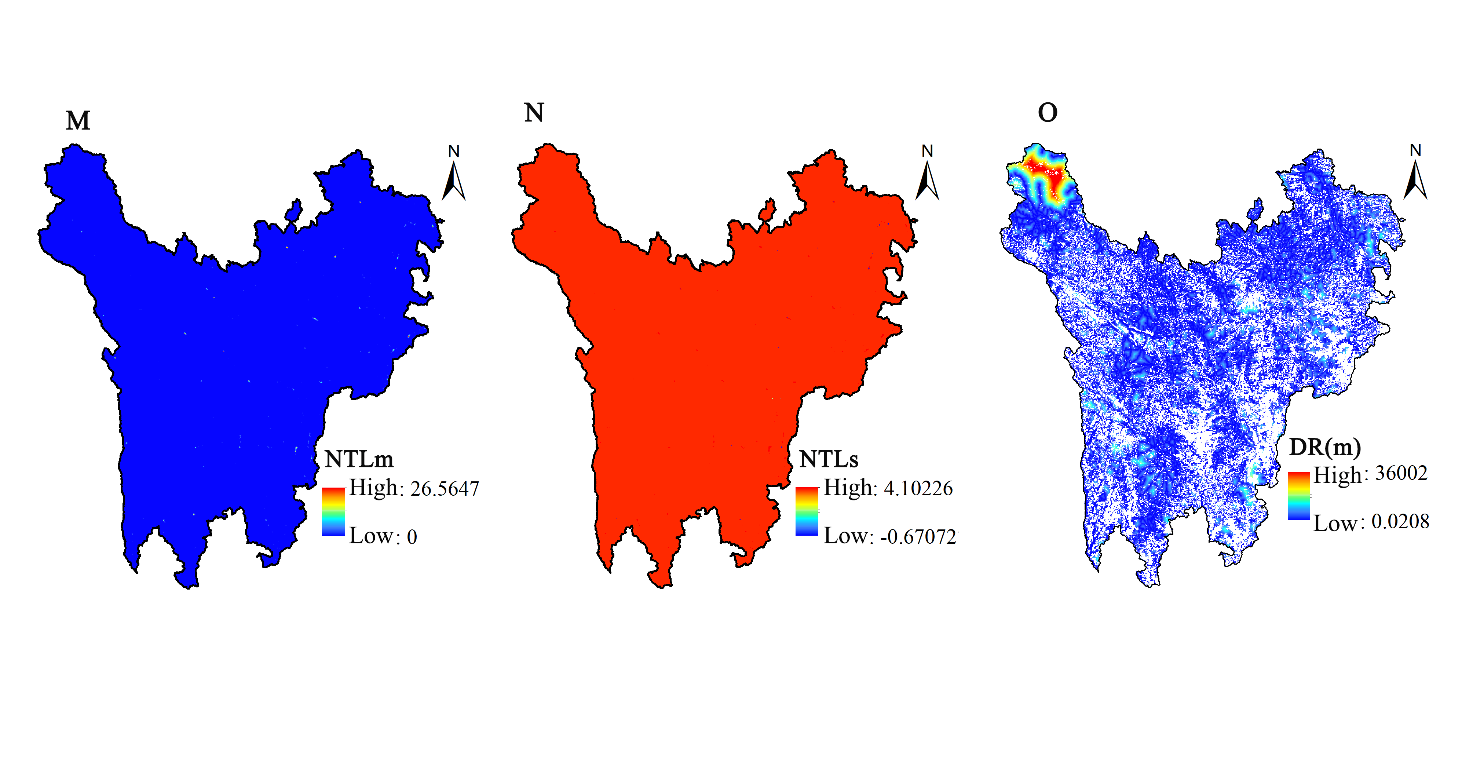

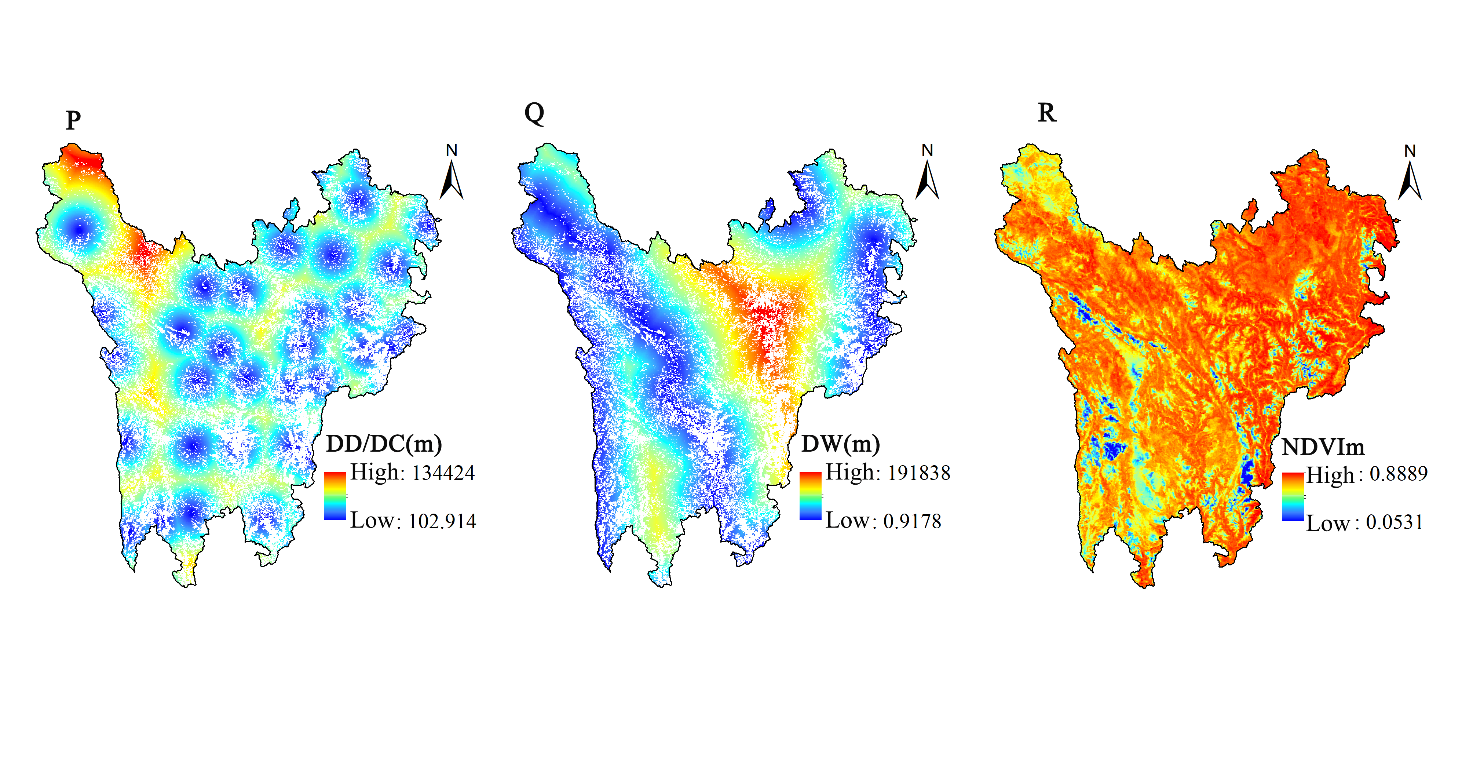


**FIGURE S1** Spatial distribution of the potential driving factors.**(A)**Clay(%). Proportion of clay. **(B)**Sand(%).Proportion of sand. **(C)**Silt(%).Proportion of silt. **(D)** Elevation (m). Elevation. **(E)** Slope(°).Slope. **(F)** Livestock(cow unit). Mean value of the number of livestock (2005-2018). **(G)**ETs. Slope of annual potential evaporation change (2005-2018). **(H)**ETm(mm). Mean value of annual potential evaporation (2005-2018). **(I)**Tm(℃). Mean value of annual temperature (2005-2018). **(J)**Ts. Slope of annual temperature change (2005-2018). **(K)** Pm(mm). Mean value of annual precipitation (2005-2018). **(L)**Ps. Slope of annual precipitation (2005-2018). **(M)**NTLm. Mean value of annual nighttime light(2005-2018). **(N)** NTLs. Slope of annual nighttime light change (2005–2018). **(O)**DR(m). The distance between each grid and its nearest road. **(P)**DD/DC(m). The distance between each grid and its nearest administrative center. **(Q)**DW(m). The distance between each grid and its nearest water body. **(R)**NDVIm. Mean value of annual Normalized Difference Vegetation Index (2005–2018)

# Supplementary Material 2

Accroding to the frequency ratio of grassland degradation and impact factors in each interval, we merged the intervals with similar frequency ratios, the result of classes as shown in Table S1.

**TABLE S1** Discretization results of BN model variables.

| Variable type | Variable | Code of level | | | | |
| --- | --- | --- | --- | --- | --- | --- |
|  |  | Lowest | Low | Medium | High | Highest |
| Topographic | DEM(mm) | 3200~3800 | 2600~3200&3800~4400 | 1400~2600&4400~5000 | <1400 | >5000 |
|  | Slope(°) |  | <5 | 5~10 | 10~25 | >25 |
| Soil | Clay(%) |  | 10~20 | 20~30&>=40 | 30~40 | <10 |
|  | Sand(%) |  | 40~80 | <40 | >80 |  |
|  | Silt(%) |  | 20~40 | >40 | <20 |  |
| Climatic | Tm(℃) | 2~6 | >6 | -2~2 | -4~-2 | <-4 |
|  | Ts |  | >0.04 | -0.04~-0.02 | <-0.04 | -0.02~0.03 |
|  | ETm(mm) |  | 700~1000&1100~1200 | 500~700&1000~1100&>1200 | 400~500 | <400 |
|  | ETs |  | <-0.4&>0.2 | -0.4~-0.3&0~0.2 | -0.3~-0.2&-0.1~0 | -0.2~-0.1 |
|  | Pm(mm) |  | 640~740 | <490&740~790&  890~990 | 790~890&990~1040 | >1040 |
|  | Ps |  | 0~4 | -6~0&4~8 | >8 | <-6 |
| Social and economic | Lm(cow unit) |  | 300000~400000 | 100000~300000 | <100000&400000~600000 | >600000 |
|  | DW(m) |  | 1500~3000 | 3000~7500 | <1500 | >7500 |
|  | DR(m) |  | >30000 | <5000&25000~30000 | 20000~25000 | 5000~20000 |
| others | NDVIm |  | >0.6 | 0.4~0.6 | 0.2~0.4 | <0.2 |
| Target variable | NDVIs |  | Decrease | Increase |  |  |
|  | SE |  | YES | NO |  |  |
|  | Grassland degradation |  | YES | NO |  |  |

# Supplementary Material 3


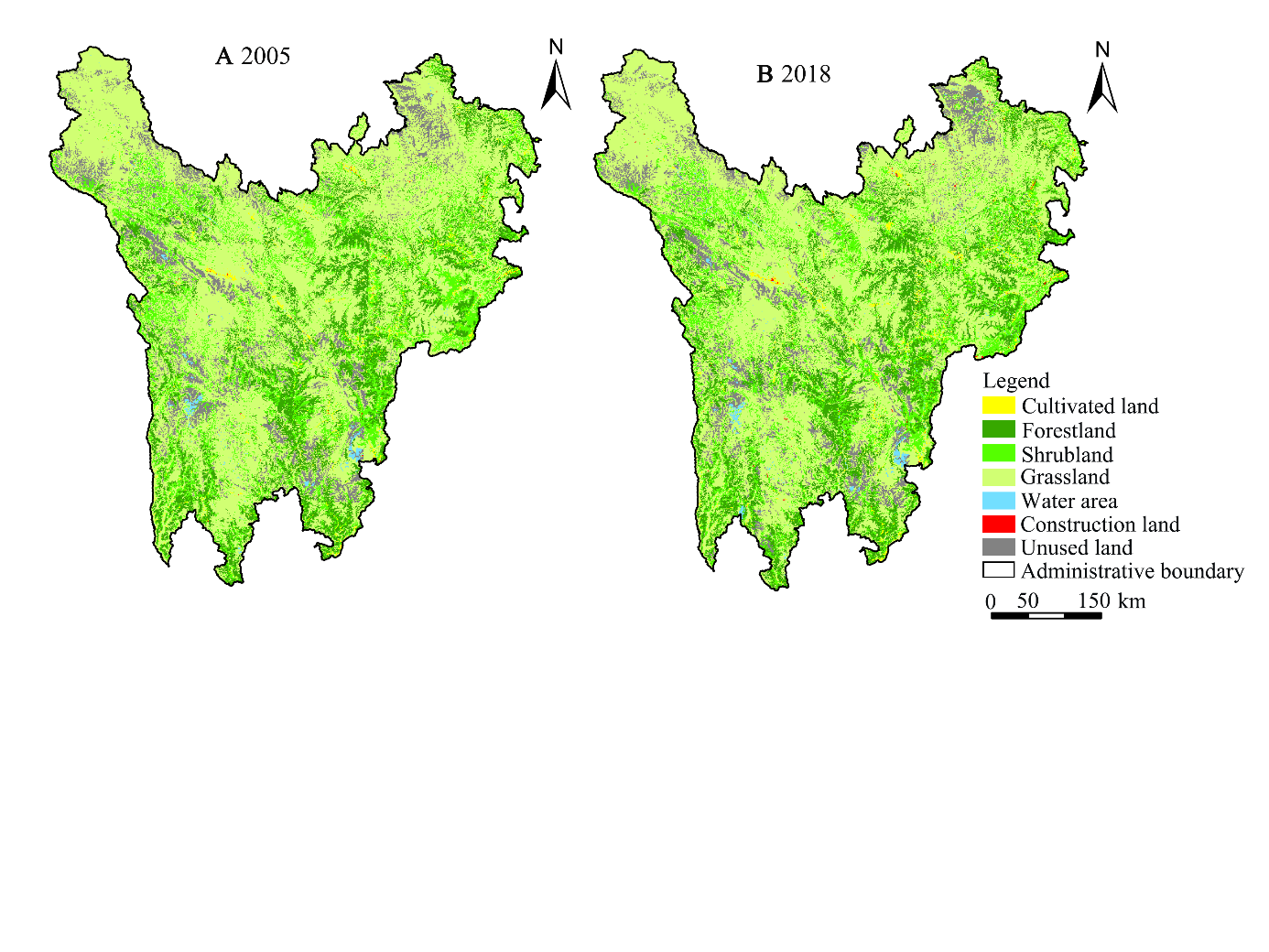


**FIGURE S2** Land use in 2005 and 2018. **(A)** Land use in 2005. **(B)** Land use in 2018.

In this study, land use data was divided into 7 categories, including cultivated land,forestland, shrubland,grassland, water area, construction land and unsed land. Shrub-encroached grasslands were identified by overlay analysis.

# References:

Chen, Y., Wang, W., Guan, Y., Liu, F., Zhang, Y., Du, J., et al. (2020). An integrated approach for risk assessment of rangeland degradation: A case study in Burqin County, Xinjiang, China. Ecol. Indic. 113, 106203. doi: 10.1016/j.ecolind.2020.106203

Eddy, I. M. S., Gergel, S. E., Coops, N. C., Henebry, G. M., Levine, J., Zerriffi, H., et al. (2017). Integrating remote sensing and local ecological knowledge to monitor rangeland dynamics. Ecol. Indic. 82, 106-116. doi: 10.1016/j.ecolind.2017.06.033

Liu, Y., Zhang, Z., Tong, L., Khalifa, M., Wang, Q., Gang, C., et al. (2019). Assessing the effects of climate variation and human activities on grassland degradation and restoration across the globe. Ecol. Indic. 106, 105504. doi: 10.1016/j.ecolind.2019.105504

Sha, Z., Zhong, J., Bai, Y., Tan, X., Li, J. (2016). Spatio-temporal patterns of satellite-derived grassland vegetation phenology from 1998 to 2012 in Inner Mongolia, China. J. Arid Land 8, 462-477. doi: 10.1007/s40333-016-0121-9

Zhou, W., Yang, H., Huang, L., Chen, C., Lin, X., Hu, Z., et al. (2017). Grassland degradation remote sensing monitoring and driving factors quantitative assessment in China from 1982 to 2010. Ecol. Indic. 83, 303-313. doi: 10.1016/j.ecolind.2017.08.019

**
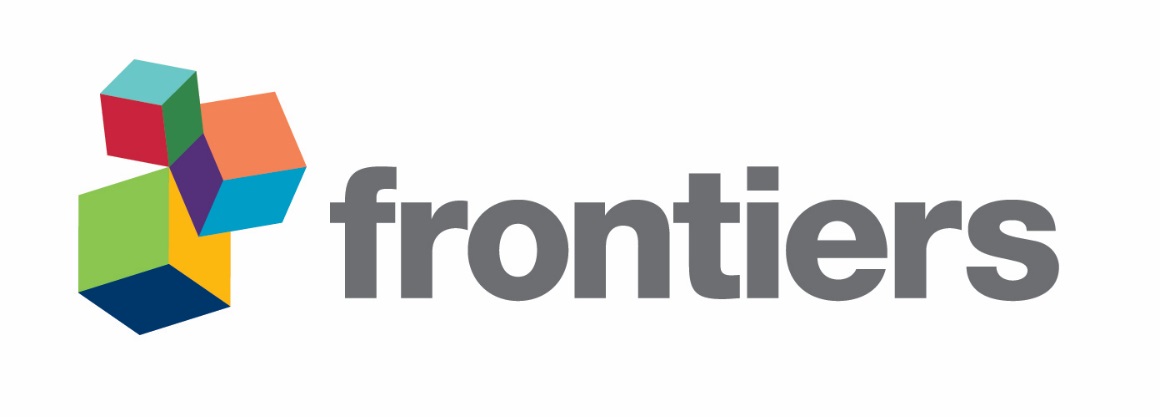
**
